# The role of ionotropic glutamate receptors in Alzheimer's disease: A scientometric analysis

**DOI:** 10.1177/13872877261423577

**Published:** 2026-03-03

**Authors:** Yunsheng Liu, Rongde Zhong, Qian Li, Yuyan Wang, Jinfang Zhang, Zengwei Kou

**Affiliations:** 1 Cancer Center, Shenzhen Hospital (Futian) of Guangzhou University of Chinese Medicine, Shenzhen, China; 2 Department of Neurosurgery, Institute of Translational Medicine, Shenzhen Second People's Hospital/the First Affiliated Hospital of Shenzhen University Health Science Center, Shenzhen, China; 3 Department of Laboratory Medicine and Pathobiology, Temerty Faculty of Medicine, 7938 University of Toronto, Toronto, ON, Canada

**Keywords:** Alzheimer's disease, bibliometric analysis, ionotropic glutamate receptors, neurodegeneration, research trends

## Abstract

**Background:**

Alzheimer's disease (AD) is a progressive neurodegenerative disorder characterized by amyloid-β plaques, neurofibrillary tangles, and synaptic dysfunction. Dysregulation of ionotropic glutamate receptors (iGluRs), including NMDA, AMPA, and kainate receptors, contributes to excitotoxicity, synaptic impairment, and cognitive decline, underscoring their therapeutic potential.

**Objective:**

This study aimed to conduct a comprehensive bibliometric analysis of iGluR research in AD from January 1, 1986 to August 23, 2025.

**Methods:**

We systematically searched and analyzed the publications related to iGluRs in AD from PubMed, Web of Science, and Scopus using CiteSpace, VOSviewer, and Bibliometrix. Metrics included publication volume, citation impact, international collaborations, keyword co-occurrence, and burst detection.

**Results:**

A total of 4810 papers were identified for analysis. The most prolific country, institution, journal, and author were the United States, Harvard University system, *Journal of Neurochemistry*, and Lipton SA, respectively. Research has evolved from NMDAR dysfunction and Aβ toxicity to clinical applications, such as memantine therapy, with recent trends focusing on AMPAR modulation and neuroprotection. Emerging researchers from China have demonstrated rapid growth. Keyword analysis reflected a sustained interest in molecular mechanisms and an increasing emphasis on clinical translation.

**Conclusions:**

This study delineates the evolution of iGluR research in AD from mechanistic insights to therapeutic innovation. While NMDARs remain central, future efforts should prioritize understudied targets like AMPARs and KARs, and leverage emerging technologies, such as cryo-electron microscopy, single-cell sequencing, and artificial intelligence, to refine therapeutic strategies and facilitate personalized medicine. It highlights targeted iGluR modulation as a promising therapeutic avenue for combating AD.

## Graphical abstract

**Graphical Abstract:**
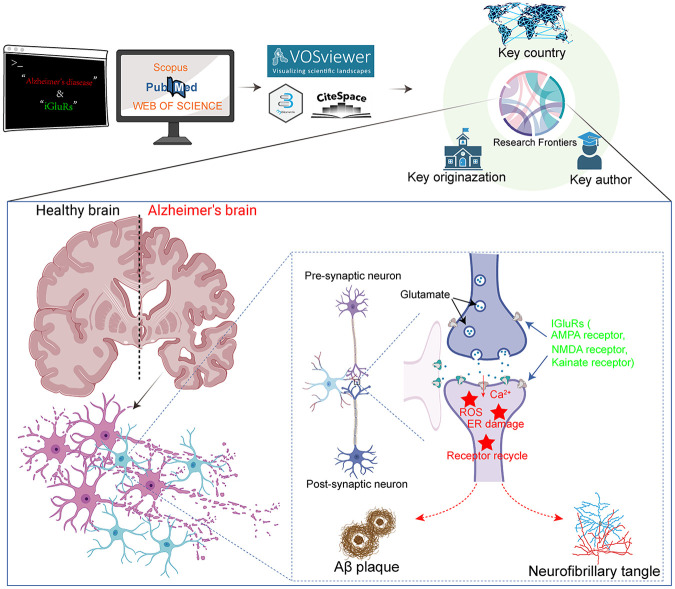
This is a visual representation of the abstract.

## Introduction

Alzheimer's disease (AD), the most prevalent neurodegenerative dementia, severely affects the health of the global elderly population through progressive cognitive decline.^1^ Its pathophysiology manifests as extracellular amyloid-β (Aβ) plaques, resulting from dysregulated amyloid-β protein precursor (AβPP) processing and intracellular neurofibrillary tangles containing hyperphosphorylated tau proteins. These pathologies coincide with synaptic loss, neuroinflammation, and oxidative stress, collectively disrupting neural circuits in memory-critical regions, such as the hippocampus and cortex. The socioeconomic burden of AD is monumental, with 55 million current cases projected to be tripled by 2050.^2^ The annual global costs surpass $1 trillion,^3^ encompassing medical care, productivity losses, and unpaid family caregiving. At the same time, it brings huge psychological and therapeutic and nursing pressure to medical staff, intensifying the social crisis. However, effective preventive and therapeutic measures for AD are currently lacking. The limited number of clinically available drugs can only partially alleviate symptoms but fail to halt or even slow the pathological progression of the disease. Therefore, there is an urgent need to identify key molecules that can target the core pathological mechanisms underlying AD.

Among the many hypotheses proposed to explain the onset and progression of AD, one significant focus involves the dysfunction of ionotropic glutamate receptors (iGluRs), which play a crucial role in excitatory neurotransmission in the central nervous system.^4^ Glutamate, the primary excitatory neurotransmitter in the brain, exerts its effects through both metabotropic and ionotropic receptors.^5^ iGluRs are pharmacologically categorized into three main subtypes: AMPA (alpha-amino-3-hydroxy-5-methyl-4-isoxazolepropionic acid) receptors (AMPARs), N-methyl-D-aspartate receptors (NMDARs), and kainate receptors (KARs).^4,6–9^ These receptors are tetrameric ion channels that allow the influx of sodium and/or calcium ions upon activation, leading to rapid changes in the membrane potential and subsequent activation of Ca_2+_-dependent intracellular signaling pathways. In the context of AD pathology, Aβ peptides have been shown to dysregulate NMDAR function, which can trigger a cascade involving metabotropic glutamate receptors and disrupt synaptic plasticity.^10^ This understanding led to the development and clinical approval of memantine, an NMDAR pore blocker, by the FDA in 2003.^11^ AMPAR, known for their high Ca^2+^ permeability and rapid activation, have also been implicated in excitotoxicity. Dysregulation of AMPAR expression and function, particularly its upregulation, is believed to contribute to glutamate-mediated neuronal injury.^12^ Several studies using AD models have demonstrated that reducing AMPAR activity can alleviate disease progression.^13–15^ Additionally, KARs have been implicated in AβPP processing in astrocytes.^16^

Despite these findings, research on iGluRs in AD remains dispersed across various molecular pathways, therapeutic targets and experimental models. This field encompasses a vast and complex body of literature, making it challenging for researchers to obtain a comprehensive understanding in a short period of time. To address this gap, we performed a bibliometric analysis to systematically map and evaluate the evolution, trends, and scientific influence of research on iGluRs in AD. Using publications retrieved from PubMed, Web of Science, and Scopus, we analyzed over 4946 papers published between 1986 and 2025. These articles were authored by more than 13,000 researchers, primarily affiliated with institutions in the United States (U.S.), China, and other countries across Europe, Asia, and the Americas, particularly the University of California system. The majority of publications appeared in journals focusing on molecular neurobiology, such as the *Journal of Neurochemistry*. Research hotspots have shifted over time, from early studies on molecular mechanisms in animal models to the development of iGluR-targeting drugs like memantine, which spurred a surge of clinical trials from 1999 to 2004. In recent years, attention has turned to preclinical screening of small molecules targeting iGluRs and the elucidation of their neuroprotective mechanisms.

Through this comprehensive bibliometric approach, we aimed to provide a visual and quantitative understanding of the structure and dynamics of iGluR-related AD research. This study not only highlights the key contributors and thematic evolution of the field but also serves as a valuable resource for guiding future investigations.

## Methods

### Data source and search

Based on previously published research between January 1, 1986, and August 23, 2025, we used the Web of Science, PubMed, and Scopus databases to collect data.^17–20^

For the Web of Science, we employed the following search query:

TS = (“ionotropic glutamate receptor” OR “AMPA receptor” OR “kainate receptor” OR “NMDA receptor” OR GRIA OR GRIK OR GRIN OR GluN* OR GluA* OR GluK*) AND TS = (“Alzheimer's disease” OR “Alzheimer disease” OR “dementia, Alzheimer type” OR “Alzheimer Type Dementia (ATD)” OR “Alzheimer syndrome” OR “Alzheimer” OR “Alzheimer's dementia” OR “dementia, Alzheimer”)

For the PubMed, we employed the following search query:

((“ionotropic glutamate receptor"[Title/Abstract] OR “AMPA receptor"[Title/Abstract] OR “kainate receptor"[Title/Abstract] OR “NMDA receptor"[Title/Abstract] OR “GRIA"[Title/Abstract] OR “GRIK"[Title/Abstract] OR “GRIN"[Title/Abstract] OR “GluN*"[Title/Abstract] OR “GluA*"[Title/Abstract] OR “GluK*"[Title/Abstract]) AND (“Alzheimer's disease"[Title/Abstract] OR “Alzheimer disease"[Title/Abstract] OR “dementia, Alzheimer type"[Title/Abstract] OR “Alzheimer Type Dementia (ATD)"[Title/Abstract] OR “Alzheimer syndrome"[Title/Abstract] OR “Alzheimer"[Title/Abstract] OR “Alzheimer's dementia"[Title/Abstract] OR “dementia, Alzheimer"[Title/Abstract])).

For the Scopus, we employed the following search query:

TITLE-ABS(“ionotropic glutamate receptor” OR “AMPA receptor” OR “kainate receptor” OR “NMDA receptor” OR GRIA OR GRIK OR GRIN OR GluN* OR GluA* OR GluK*) AND TITLE-ABS(“Alzheimer's disease” OR “Alzheimer disease” OR “dementia, Alzheimer type” OR “Alzheimer Type Dementia (ATD)” OR “Alzheimer syndrome” OR “Alzheimer” OR “Alzheimer's dementia” OR “dementia, Alzheimer”)

### Data analysis

The PubMed dataset was exported in .nbib format and subsequently converted into the Web of Science .txt format using CiteSpace.^21^ Data from Scopus were obtained in .csv format and converted into the Web of Science format within CiteSpace. Following format harmonization, the three datasets were merged into a single composite database, and duplicate entries were systematically identified and removed. The consolidated integrated database was then analyzed using CiteSpace, VOSviewer,^22^ and Bibliometrix^23^ to facilitate a comprehensive exploration and visualization of research trends, emerging hotspots, and key contributors within the field of iGluR research in Alzheimer's disease. Selected visual representations were generated using GraphPad Prism v.8.0, and comparisons between two groups were conducted using Student's t-test.

## Results

### Key discoveries and milestones in AD research

Before analyzing the relationship between AD and iGluRs, we summarized the landmark events and milestones in AD research. Since Alois Alzheimer first described the disease in 1906 as “a peculiar severe disease process of the cerebral cortex”, there has been little significant progress for nearly half a century. It was only in the 1950s-1960s that AD was firmly established as a distinct neurodegenerative disorder.^24^

Over subsequent decades, multiple pathogenic hypotheses have been proposed to explain AD, including the cholinergic hypothesis,^25^ genetic models driven by AβPP mutations^26^ and presenilin mutations (PSEN1/PSEN2),^27^ and the amyloid cascade hypothesis.^28^ Additional mechanistic frameworks have implicated tau pathology, neuroinflammation, vascular contributions, and synaptic dysfunction.^29^ In line with these hypotheses, a range of therapeutic agents have been developed and approved: cholinesterase inhibitors such as donepezil, rivastigmine, and galantamine for symptomatic treatment; the NMDA receptor modulator memantine (Namenda); and more recently, anti-amyloid monoclonal antibodies including aducanumab (Aduhelm), lecanemab (Leqembi) and donanemab (Kisunla) as disease-modifying agents for biomarker-positive early AD.^30^

**Figure 1.**
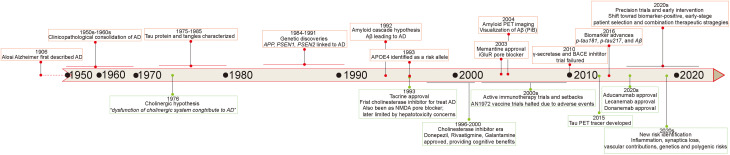
Key discoveries and milestones in AD research.

Concurrently, substantial efforts have focused on earlier and more accurate diagnosis. Advances include amyloid and tau PET tracers (e.g., PiB for amyloid; tracers such as flortaucipir for tau) and fluid biomarkers such as phospho-tau (p-tau181, p-tau217) and plasma Aβ assays.^31^ Nevertheless, AD etiology is complex and heterogeneous: contemporary evidence supports a multifactorial model in which genetic risk, inflammation, synaptic loss, and vascular pathology all contribute.^32^ Accordingly, the field is shifting toward earlier, biomarker-guided, precision and combination therapeutic strategies (Figure 1). This timeline underscores the multifaceted nature of AD research, evolving from initial pathological descriptions to biomarker-driven therapeutic strategies.

### Analysis of publication output and temporal trends

**Figure 2.**
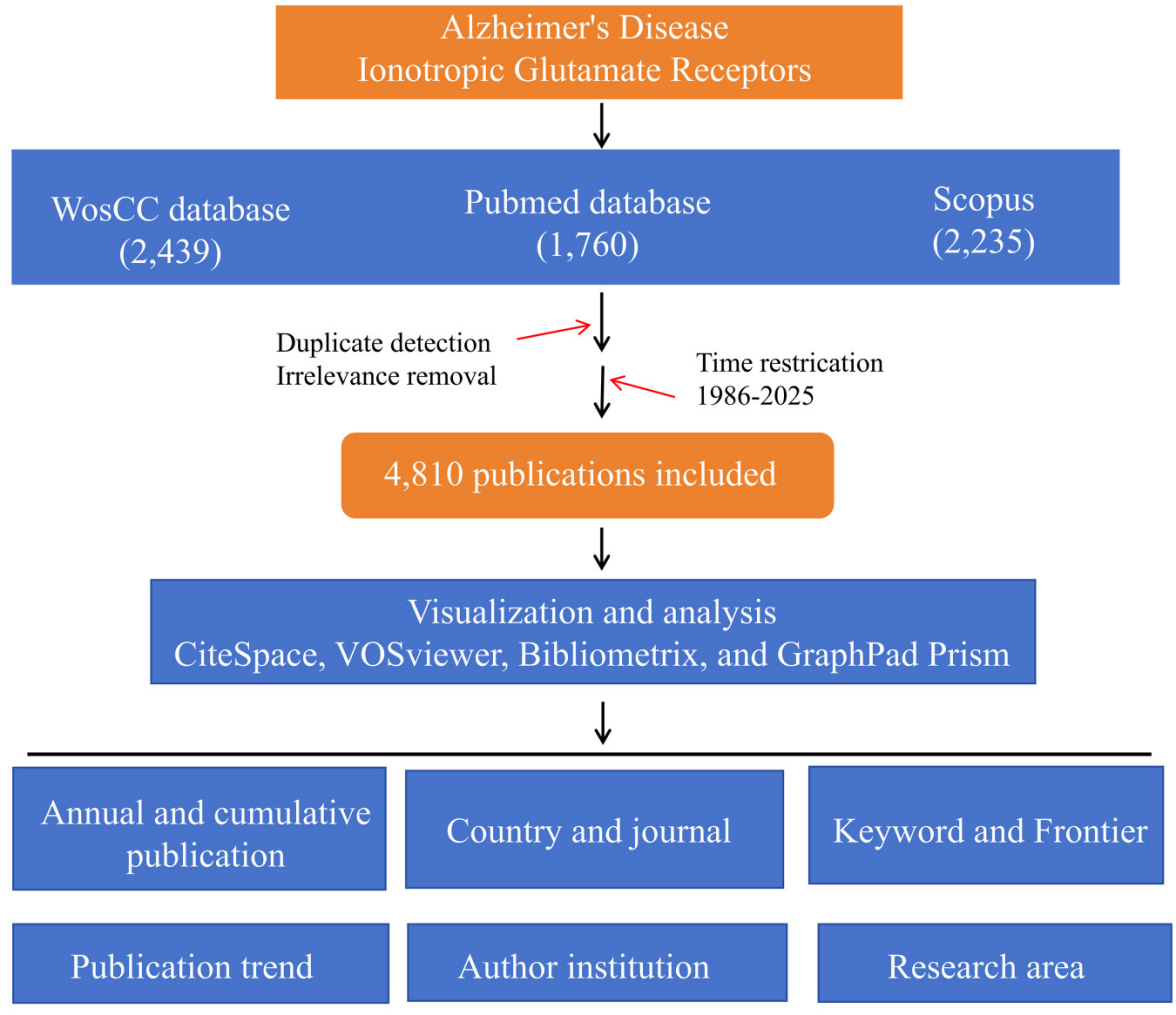
Flow chart of literature search and screening.

**Figure 3.**
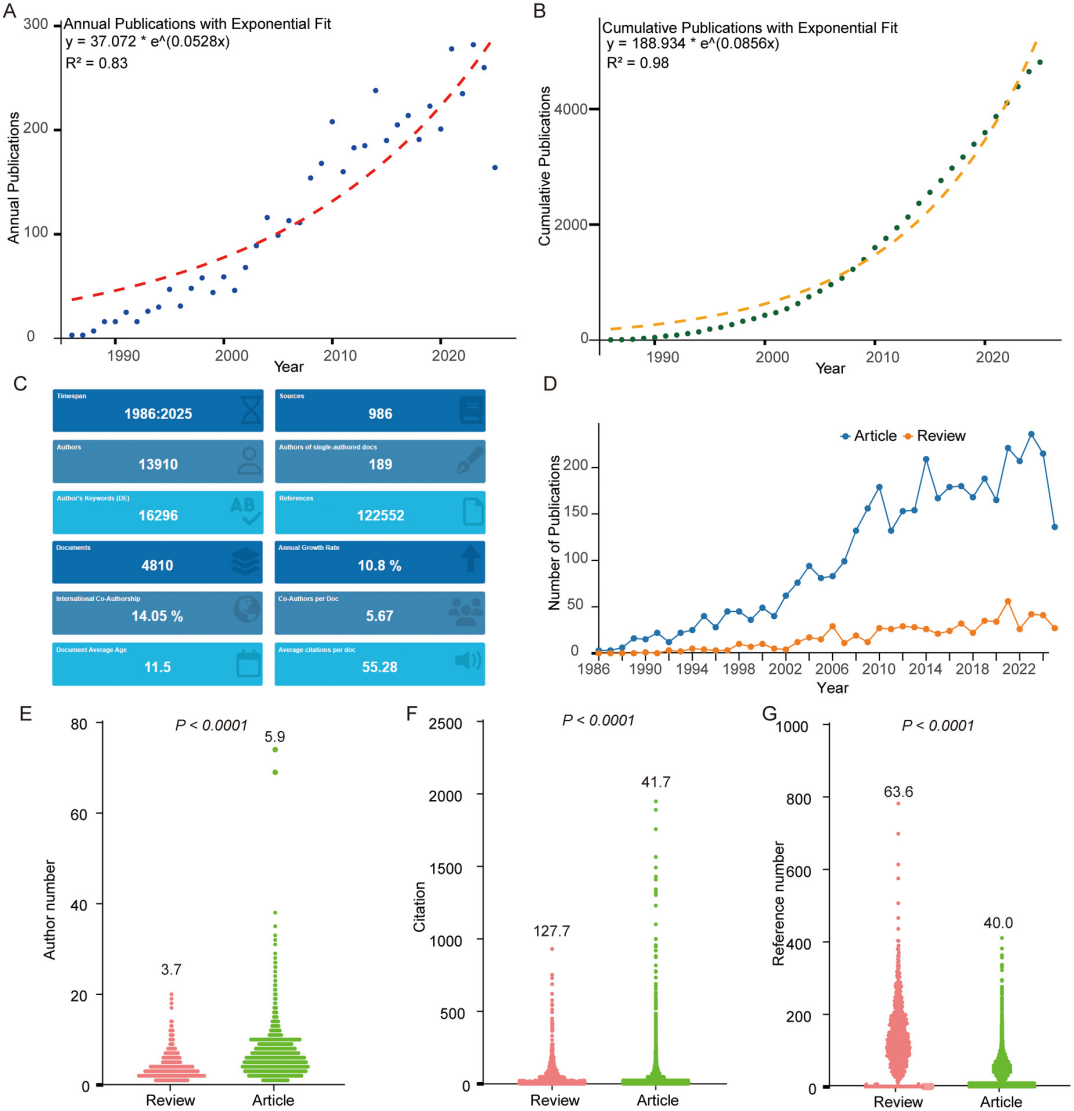
Overall trends in iGluR and Alzheimer's disease research. (A, B) Annual and cumulative publication counts over time, with fitted mathematical growth model. (C) General bibliometric information for all included articles. (D) Annual publication trends of original articles versus review articles. (E-G) Comparative analysis of (original) research articles and reviews in terms of the number of authors, references, and citations.

Using the personalized Boolean search terms, we retrieved 2439 articles from Web of Science, 1760 from PubMed, and 2235 from Scopus. Each author independently screened the titles and abstracts to ensure relevance to the research theme. The collected records were first standardized and merged using CiteSpace. After deduplication and applying the publication time constraints, 4810 publications were recognized by Bibliometrix. (Figures 2 and 3C).

It is worth noting that Bibliometrix was used for statistics-related results, while CiteSpace was employed to analyze top-cited authors, institutions, and temporal trends. The earliest article identified was an original study published in *Neuroscience Research* journal titled “Plasticity in hippocampal excitatory amino acid receptors in Alzheimer's disease”. Since then, the annual publication output has grown steadily, with a compound annual growth rate of 10.8% (because publications from 2025 are still being updated, this value is likely to be underestimated). Unlike the plateau or post-peak decline observed in research of iGluRs, such as AMPAR, NMDAR or KAR research,^17,19,20^ this field has shown consistent expansion.

Both the annual and cumulative publication counts demonstrated a rapid growth trend. Notably, cumulative publications fit well with an exponential growth model with R^2^ = 0.98 (Figure 3A, B). The articles received an average of 55.28 citations (Figure 3C), which is substantially higher than that reported in AMPAR,^19^ NMDAR,^17^ or KAR research.^20^ Importantly, since 1986, the number of (original) research articles has consistently exceeded that of review articles, and this disparity became even more pronounced after the 2000s (Figure 3D). From a bibliometric perspective, reviews generally involved fewer authors but contained more references and were more frequently cited than (original) research articles (Figure 3E-G).

### The evolving landscape of national and institutional research output

**Figure 4.**
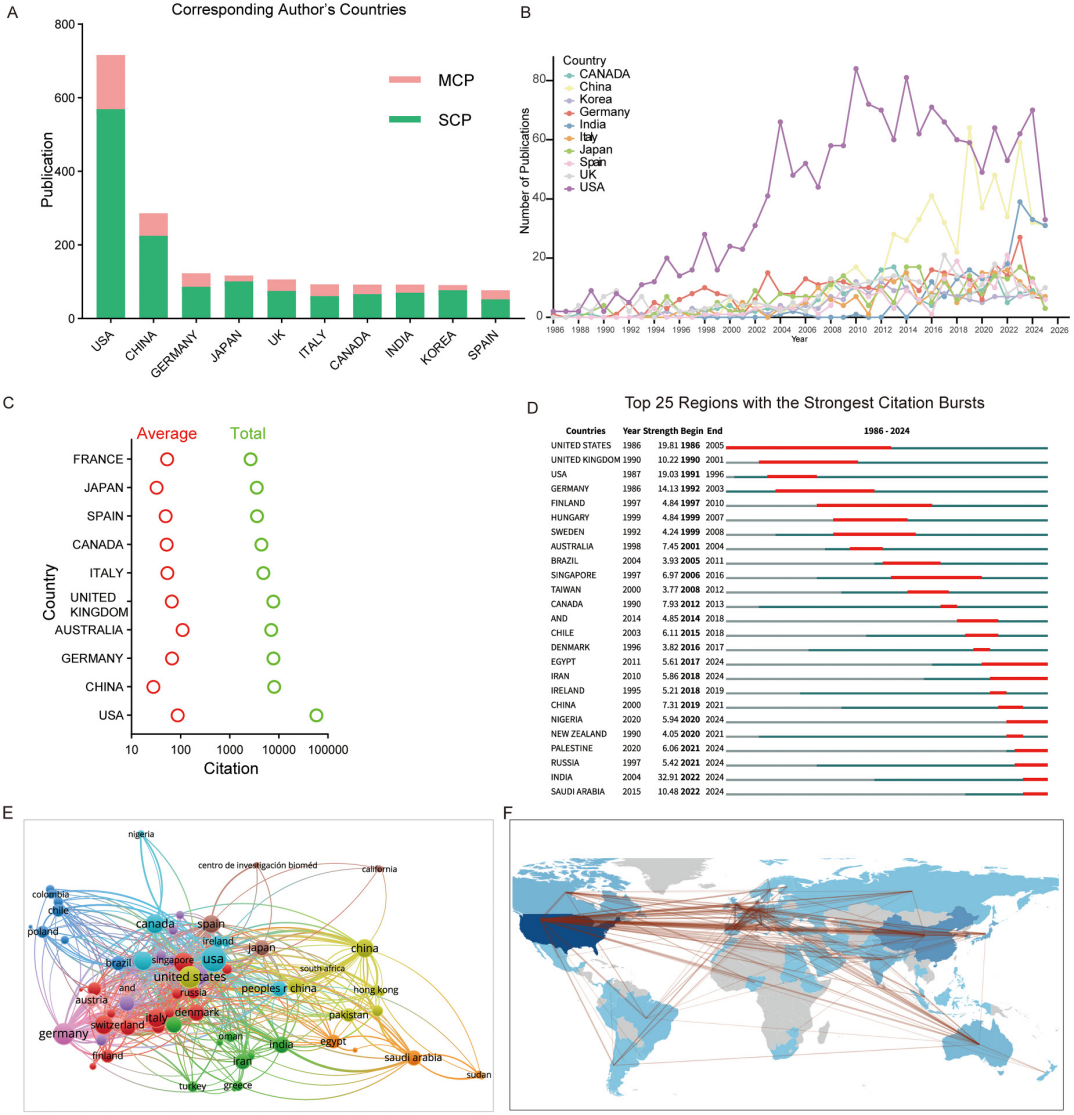
The output and cooperation of countries and institutions. (A) Top 10 countries with the most published articles. The red column shows the proportion of multiple countries’ publications (MCP), and the green column shows the proportion of single countries’ publications (SCP). (B) The top ten countries in iGluRs and AD research production over time. (C) Top 10 countries with the highest average and total citation counts. (D) Top 25 countries with the strongest citation bursts. (E) Overlay network of countries engaged in iGluRs and AD research. (F) World map of countries and regional cooperation.

An analysis of the corresponding authors’ affiliations revealed that the U.S. and China led the publication output. The U.S. ranked first with 670 publications, followed by China with 290, jointly accounting for more articles than the countries ranked 3rd to 8th combined (Germany to India) (Figure 4A).

Examining the publication trends over time in the top 10 countries revealed that the U.S. has maintained a leading position since 1992, with a sharp increase around 1999 that left countries far behind. In contrast, China published its first related article in 2000 and surpassed Germany to take second place in 2010. Since then, China's annual growth rate has outpaced that of the U.S. Other countries exhibited similar growth trends (Figure 4B). We further assessed each country's impact based on the total and average citations. As expected, the U.S. ranked highest in both total and per-paper citation counts. Although China ranked second in total publications, its total citations were comparable to those of other countries, and its average citation per article (28.0) was significantly lower than that of the U.S. (87.6) (Figure 4C).

These patterns were also reflected in the top 25 regions with the strongest citation bursts. Countries with high citation bursts during their peak periods included the U.S., the UK, and Germany (citation strength >10), while China showed a much lower strength of 7.31, indicating substantial room for improvement given its publication volume. Notably, emerging countries such as India and Saudi Arabia, demonstrated strong bursts, especially India, which, since 2022, has shown a citation strength of 32.91 (Figure 4D).

Among the top 10 publishing countries, European nations like Italy and Spain had the highest proportion of internationally co-authored papers, exceeding 31% (Figure 4A). Network and geographic analyses of international collaborations revealed that European countries formed the core of global research cooperation, extending to the Americas, Asia, and Oceania (Figure 4E, F). Singapore emerged as a central node in the global collaboration network, whereas East Asian countries, such as Japan, China (including Hong Kong), and Pakistan, appeared to form their own collaboration clusters extending toward Egypt. South Asian countries like India had strong ties with Iran, Turkey, and Greece. Interestingly, Saudi Arabia formed an independent cluster, primarily collaborating with Hong Kong and Sudan. In South America, Colombia and Chile also showed trends in forming their own collaboration hubs. Overall, these findings illustrate a clear global leadership by the U.S. in both research output and academic influence within the iGluR-AD field, while China has emerged as a rapidly growing contributor, though its scientific impact, as reflected by citation metrics, remains comparatively modest. The persistent gap between publication volume and citation impact for China suggests a need to enhance research quality and international visibility. Moreover, the distinct collaboration patterns, ranging from the tightly knit European network to the emerging regional hubs in Asia and South America, highlight the increasingly decentralized yet interconnected nature of global scientific cooperation in this domain.

### Output dynamics and evolving influence of affiliations, journals, and researchers

**Figure 5.**
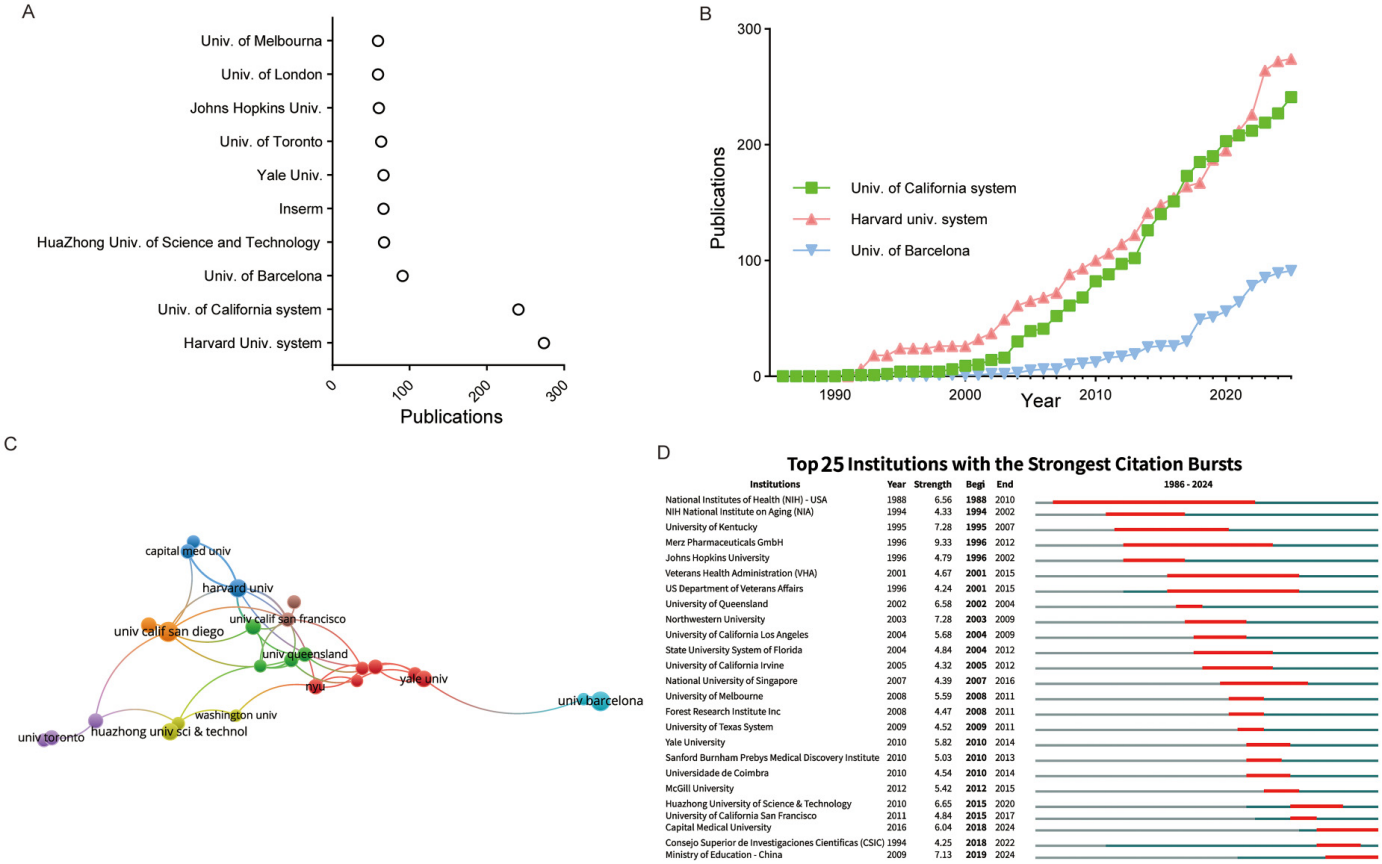
Trends in author affiliations engaged in iGluRs and AD research. (A) Dot plot of the top 10 universities or research institutions in iGluRs and AD research. (B) The top three universities in iGluRs and AD research production over time. (C) Overlay network of institutions engaged in iGluRs and AD research. (D) Top 25 Institutions with the strongest citation bursts.

The Harvard University system ranked first globally in terms of the number of publications in this field, with 274 articles published. It was followed by University of California system (241 articles). The University of Barcelona ranked third place with 91 articles. Institutions ranked 4th to 10th published between 67 and 59 articles (Figure 5A). These findings underscore the dominance of a few elite institutions in shaping the research landscape. Temporal analysis of the top four institutions showed that the Harvard University system maintained a leading position between 1992–2015. From 2016 to 2019, the University of California system briefly surpassed the Harvard University system to hold the top position in publication output. The University of Barcelona consistently maintained its third-place ranking (Figure 5B).

The co-authorship map of institutions revealed that several major universities formed collaboration clusters centered around themselves. At the geometric center of the network was the University of Queensland, which showed strong collaborative ties with institutions such as New York University and the University of California, San Francisco. Slightly further outward were other prominent institutions, including Yale University, Harvard University, University of California, San Diego, and Washington University, which also served as core nodes. On the periphery, the University of Barcelona was positioned, maintaining close collaborations with Yale University. Two Chinese universities, Capital Medical University and Huazhong University of Science and Technology, were also located at relatively peripheral cores, collaborating mainly with Harvard University and the University of Toronto, respectively (Figure 5C). These trends not only reflect the historical dominance of North American institutions in foundational iGluR-AD research but also highlight the recent emergence of Chinese institutions as influential contributors, signaling a gradual yet significant shift in the global research landscape.

The citation burst analysis of the top 25 institutions revealed dynamic shifts in active contributors. Traditional North American institutions, such as the NIH, Johns Hopkins University, and Northwestern University, were the most active in the 1990s and the early 2000s. More recent contributors include Yale University and McGill University. Huazhong University of Science and Technology became active after 2015, which is consistent with earlier observations (Figure 5D). The emergence of new institutional actors, particularly from China, signals a shifting geography of scientific influence, though their late entry and peripheral network positions suggest they are still building capacity for sustained impact.

**Figure 6.**
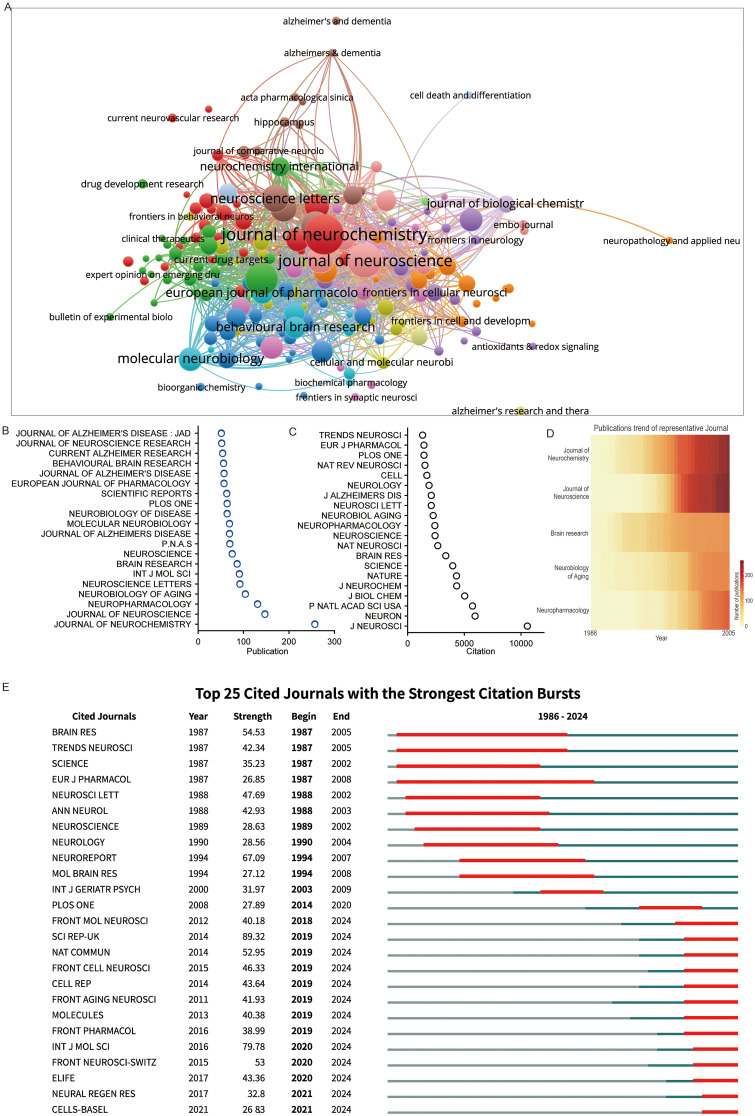
Trends in journals that published iGluRs and AD research. (A) Overlay network of journals engaged in iGluRs and AD research. (B) Dot plot of the top 20 most published journals on the iGluRs and AD. (C) Dot plot of the top 20 most impactful journals on iGluRs and AD. (D) The trajectory of research on iGluRs and AD, as reflected in five prominent journals. (E) Top 25 journals with the strongest citation bursts.

A total of 986 journals were identified in the field (Figure 3C). Journal citation network analysis revealed a core cluster centered around *Journal of Neurochemistry* and *Journal of Neuroscience*, also including *Neuroscience Letters*, *European Journal of Pharmacology*, *Behavioral Brain Research*, and *Journal of Biological Chemistry*. Several Frontiers journals, such as *Frontiers in Cell and Developmental Biology*, *Frontiers in Synaptic Neuroscience*, and *Frontiers in Cellular Neuroscience*, were also well-represented. AD-focused journals such as *Alzheimer's & Dementia* and *Alzheimer's Research & Therapy* formed their own clusters (Figure 6A). In terms of publication volume, *Journal of Neurochemistry* led with over 200 articles, followed by *Journal of Neuroscience* and *Neuropharmacology*. The other top journals published between 104 and 51 articles (Figure 6B). Evaluating the impact via total citations, *Journal of Neuroscience* surpassed *Journal of Neurochemistry* to become the most influential. Despite not being among the top 20 journals by publication volume, *Neuron* ranked second in terms of total citations. General high-impact journals, such as *PNAS*, *Nature*, *Science*, and *Cell*, also demonstrated a strong influence (Figure 6C).

Temporal analysis showed that *Journal of Neurochemistry* overtook *Neuroscience Letters* in 1996 and maintained the top position. The *Journal of Neuroscience* followed a similar growth pattern, albeit five years later. Around 2014, *Neurobiology of Aging* saw a temporary spike in output, reaching third place before being surpassed by *Neuropharmacology* in 2017 (Figure 6D). The top 25 cited journals list further detailed the activity periods of these journals. Earlier active journals, such as *Brain Research*, *Trends in Neurosciences*, *Science*, and *European Journal of Pharmacology*, were prominent from the 1980s to the 2000s, followed by *Neurology* and *Molecular Brain Research*, and later by open-access journals such as *PLoS One*, *Scientific Reports*, *Nature Communications*, and *ELife* (Figure 6E). Overall, the iGluR-AD research field features a dynamic journal landscape. While established neuroscience journals have historically led in output, their citation influence is increasingly rivaled by high-impact multidisciplinary journals like *Neuron*, *Nature*, and *Science*, indicating that groundbreaking work often gains visibility through broader-audience platforms. The growing role of open-access journals such as *Frontiers* and *Nature Communications* reflects a shift toward greater accessibility. However, the concentration of high-impact studies in a few selective journals raises concerns about publication bias. Future studies could assess whether this publication pattern aligns with real translational and clinical advances.

**Figure 7.**
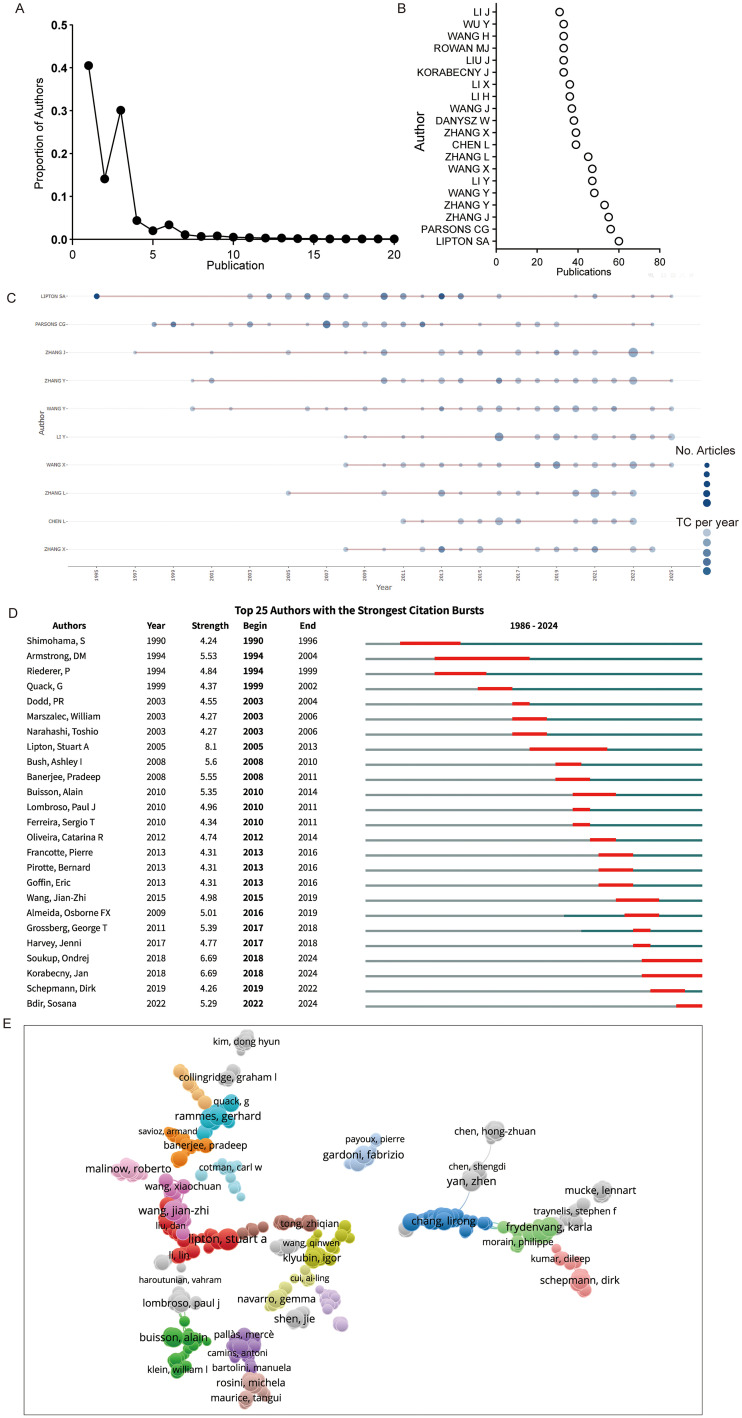
Author summary in iGluRs and AD research. (A) Author productivity according to Lotka's law. (B) Dot plot of the top 20 authors in iGluRs and AD research. (C) Top 10 authors in the field of iGluRs and AD research over time. (D) Top 25 authors with the strongest citation bursts. (E) Overlay network of authors engaged in iGluRs and AD research.

In total, 13,910 authors contributed to publications in this field, with an average of fewer than 0.5 articles per author (Figure 3C). More than 90% of the authors published fewer than five articles (Figure 7A). Leading among them is Lipton SA, who is the most prolific author with over 60 publications related to this topic and remained active for three decades. His sustained scholarly output includes numerous high-impact articles, solidifying his influence within the research community. Other long-standing prolific authors include Parsons CG and Zhang J, who have been deeply engaged in the field for over 20 years (Figure 7B, C). Notably, several emerging researchers, such as Chen L and Zhang X, have recently become active and demonstrated a rapid increase in publication output despite their shorter academic histories (Figure 7B, C).

Citation burst analysis further highlights the prominence of Lipton SA, who not only leads in publication volume but also exhibits the strongest citation burst with a strength value of 8.1. Additionally, Soukup O and Korabecny J, who began publishing in this area around 2018, showed significant recent citation momentum with burst strengths of 6.69 each (Figure 7D). The co-authorship and citation networks of key researchers displayed a multi-centric structure. A prominent author core has formed around Lipton SA, including frequent collaborators, such as Lin L and Liu D, placing this cluster at the center of the network. Other independent author groups have coalesced around figures like Buisson A, Klyubin I, Malino R, Banerjee P, Rammes G, Chang L, and Frydenvang K, indicating diversified yet structured collaboration patterns (Figure 7E). Overall, the field features a wide range of contributors with generally low output, alongside a small group of highly productive and influential authors. While pioneers like Lipton SA show long-term impact, and new researchers bring fresh perspectives, the field relies heavily on a few key authors. The concentration of citations among few authors may indicate thematic narrowness or difficulty in gaining recognition. To ensure sustainable growth, the field should develop a more distributed and robust author network.

### Research trends: keywords and hotspots

**Figure 8.**
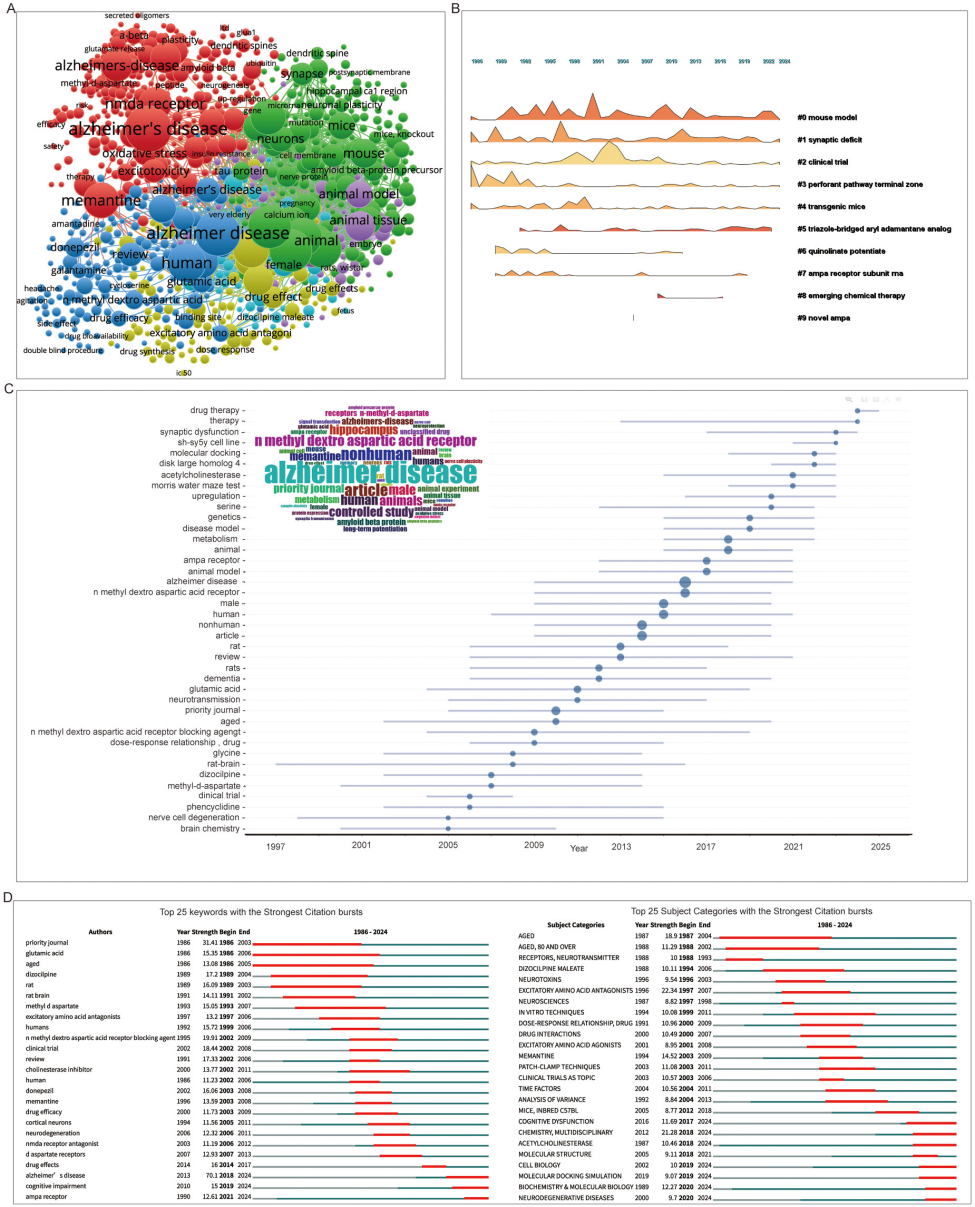
Keywords in iGluRs and AD research. (A) Overlay network of keywords engaged in iGluRs and AD research. (B, C) Evolution of keywords over time in iGluRs and AD research. The insert in (C) displays the occurrence of the keyword, with the size of the word representing the probability of occurrence. (D) Top 25 subject categories/ keywords with the strongest citation bursts.

Keyword co-occurrence analysis revealed that various formulations of “Alzheimer's disease” dominate the landscape, forming the foundation of multiple keyword clusters that together constitute a large, densely connected “round-like ball” core (Figure 8A). This core reflects the field's sustained interest in the molecular mechanisms underlying AD pathology, as evidenced by frequent terms such as NMDAR, Aβ, tau protein, and amyloid beta. In parallel, terms related to disease induced neurobiological alterations, such as LTD, dendritic spine, neuroplasticity, and oxidative stress, also appear prominently. Macro-level clinical symptoms, including headaches, are additionally represented. Intervention strategies for AD are gaining attention, with small-molecule agents such as amantadine and cycloserine showing promise. Clinical applications and pharmacological investigations are further reflected through keywords such as drug synthesis, dose response, and drug effect(s). Temporal keyword evolution analysis (Figure 8B, C) showed that foundational terms such as mouse model, animal, rats, synaptic deficit, and clinical trial have remained prevalent throughout all time periods. In contrast, recently emerging keywords include triazole-bridge aryl adamantane analog and AMPAR subunit RNA. The term novel AMPA, which was briefly active around 2005, has since faded, suggesting a transient research interest.

In terms of citation strength, keywords like NMDAR blocking agent and clinical trial rank highest among the top 25, indicating their substantial impact within the field (Figure 8D). Subject category analysis revealed that the most highly cited categories were excitatory amino acid antagonists and chemistry (Figure 8D), highlighting the field's dual focus on neuropharmacology and molecular sciences. The keyword co-occurrence and evolution analyses not only map the intellectual structure of iGluR research in AD but also reveal its thematic continuity and shifting priorities. The persistent dominance of terms related to molecular mechanisms, such as NMDAR, Aβ, and tau, underscores the field's enduring focus on understanding the fundamental pathology of AD. However, the emergence of keywords like triazole-bridge aryl adamantane analog and AMPAR subunit RNA signals a gradual shift toward targeted molecular interventions and genetic-level mechanisms, reflecting an evolving research agenda aimed at precision medicine.

A review of the top 16 most-cited original articles further underscores the central themes of this field. We found that six articles explicitly mentioned memantine, an NMDAR pore blocker, in their titles, highlighting the considerable attention given to its therapeutic potential for AD. Unsurprisingly, seven articles included “NMDA receptor” in their titles, with research primarily focusing on three aspects: (i) the therapeutic effects of memantine targeting NMDARs in AD, (ii) the impact of Aβ on NMDARs trafficking and function, and (iii) the influence of Aβ on the relative proportions of NMDAR subtypes at synapses. This also explains why Aβ appeared in the titles of 11 articles. Importantly, the role of AMPARs has not been overlooked. For example, a 2006 paper entitled “*AMPAR Removal Underlies Aβ-Induced Synaptic Depression and Dendritic Spine Loss*” investigated how AMPARs contribute to Aβ-induced synaptic dysfunction and dendritic spine loss (Table 1).

**Table 1.** Top 16 references with the strongest citation bursts.

Title	DOI	Year	Citation strength
Memantine in severe dementia: results of the 9M-best study (benefit and efficacy in severly demented patients during treatment with memantine)	10.1002/(SICI)1099-1166(199902)14:2	1999	15.6114
Memantine in Moderate-to-Severe Alzheimer's Disease	10.1056/NEJMoa013128	2003	41.4918
Neuroprotection by memantine against neurodegeneration induced by β-amyloid(1–40)	10.1016/S0006-8993(02)03731-9	2002	18.4271
Memantine Treatment in Patients With Moderate to Severe Alzheimer Disease Already Receiving Donepezil A Randomized Controlled Trial	10.1001/jama.291.3.317	2004	31.6618
The NMDA receptor antagonist memantine as a symptomatological and neuroprotective treatment for Alzheimer's disease: preclinical evidence	10.1002/gps.938	2003	24.0808
Regulation of NMDA receptor trafficking by amyloid-β	10.1038/nn1503	2005	34.0904
AMPAR Removal Underlies Aβ-Induced Synaptic Depression and Dendritic Spine Loss	10.1016/j.neuron.2006.10.035	2006	18.8191
Aβ Oligomer-Induced Aberrations in Synapse Composition, Shape, and Density Provide a Molecular Basis for Loss of Connectivity in Alzheimer's Disease	10.1523/JNEUROSCI.3501-06.2007	2007	20.2968
Aβ Oligomers Induce Neuronal Oxidative Stress through an N-Methyl-D-aspartate Receptor-dependent Mechanism That Is Blocked by the Alzheimer Drug Memantine	10.1074/jbc.M607483200	2007	16.127
Natural Oligomers of the Alzheimer Amyloid-β Protein Induce Reversible Synapse Loss by Modulating an NMDA-Type Glutamate Receptor-Dependent Signaling Pathway	10.1523/JNEUROSCI.4970-06.2007	2007	30.0231
Amyloid-β protein dimers isolated directly from Alzheimer's brains impair synaptic plasticity and memory	10.1038/nm1782	2008	26.7269
Soluble Oligomers of Amyloid β Protein Facilitate Hippocampal Long-Term Depression by Disrupting Neuronal Glutamate Uptake	10.1016/j.neuron.2009.05.012	2009	17.8353
Dendritic Function of Tau Mediates Amyloid-β Toxicity in Alzheimer's Disease Mouse Models	10.1016/j.cell.2010.06.036	2010	16.1568
Soluble Aβ Oligomers Inhibit Long-Term Potentiation through a Mechanism Involving Excessive Activation of Extrasynaptic NR2B-Containing NMDA Receptors	10.1523/JNEUROSCI.0203-11.2011	2011	27.7077
Early neuronal dysfunction by amyloid β oligomers depends on activation of NR2B-containing NMDA receptors	10.1016/j.neurobiolaging.2010.01.011	2011	15.8985
Aβ induces astrocytic glutamate release, extrasynaptic NMDA receptor activation, and synaptic loss	10.1073/pnas.1306832110	2013	16.3427

## Discussion

Since its discovery in 1906, AD has lacked effective prevention and treatment measures.^33,34^ The present bibliometric analysis provides a comprehensive overview of the research landscape concerning iGluRs in AD, highlighting key trends, contributors, and evolving themes over nearly four decades. Our study underscores the sustained growth of this field, with a compound annual growth rate of 10.8%, reflecting its critical role in understanding AD pathogenesis and therapeutic development. Below, we contextualize our findings within the current research landscape, identify gaps, and discuss the implications of our results for future investigations.

### Current research status

The study of iGluRs in AD has evolved from foundational molecular and biochemical explorations to translational and clinical research.^35,36^ The U.S. and China dominate the field, with institutions like the University of California system and Huazhong University of Science and Technology leading in publication output. Key journals, such as the *Journal of Neurochemistry* and *Journal of Neuroscience*, have been pivotal in disseminating high-impact research. Notably, NMDARs, particularly their interaction with Aβ, remain the primary focus, as evidenced by the prominence of memantine-related studies and the underrepresentation of AMPA and KARs. This reflects the field's historical emphasis on excitotoxicity and synaptic dysfunction mediated by NMDARs.

### Identified gaps and challenges

Despite significant progress, several gaps persist. First, the disproportionate focus on NMDARs and AMPARs left KARs underexplored, despite their potential roles in AD pathology. Second, while bibliometric tools like CiteSpace and VOSviewer offer robust quantitative analyses, they may overlook nuanced qualitative insights, such as the translational potential of preclinical findings. Third, although China has rapidly increased its publication volume, its research impact, measured by citations, lags behind that of the U.S. and Europe, suggesting a need for higher-quality studies and stronger international collaborations.

### Key findings and implications

Our analysis revealed several critical insights: First, Collaboration Networks: This field is characterized by decentralized, multi-centric collaborations, with prominent researchers like Lipton SA serving as hubs. This structure fosters diversity but may benefit from more interdisciplinary integration, particularly between basic science and clinical research. Second, research hotspots: Early studies focused on molecular mechanisms like the interactions between Aβ and NMDAR, while recent trends emphasize clinical translation, such as small-molecule therapeutics and neuroprotective strategies. The keyword “NMDAR blocking agent” and “clinical trial” exhibited strong citation bursts, underscoring the growing emphasis on therapeutic development. In addition, the geographical and institutional trends show that the U.S. maintains leadership in both output and impact, but China's rapid growth signals its emerging role. However, China's low average citation rate highlights the need for enhanced research quality and global engagement.

Our findings have significant research and clinical implications. For instance, the dominance of NMDAR studies validates the clinical use of memantine, while pointing to untapped opportunities in targeting AMPARs and KARs.^37–39^ Additionally, the shift toward clinical translation suggests a maturing field poised to bridge bench-to-bedside gaps.

Despite extensive research on NMDARs, particularly regarding memantine and Aβ pathology, studies on AMPARs remain underrepresented, highlighting a promising yet underexplored avenue for future investigation. Subject category analysis further confirmed the dominance of excitatory amino acid antagonists and pharmacological chemistry. Leading journals include *Journal of Neurochemistry* and *Journal of Neuroscience*, with increasing influence from open-access platforms such as *Frontiers* and *ELife*. However, top-tier journals, such as *Neuron*, *Nature*, and *Science*, have continued to validate these high-impact discoveries.

### Future directions and emerging hotspots

The following areas are poised to shape future research: mounting evidence highlights the pivotal role of NMDARs in AD pathogenesis, with abundant small-molecule modulators (e.g., pore blockers, competitive antagonists, allosteric regulators)^40–42^ representing potential drug precursors. Beyond NMDARs, targeting AMPARs and KARs may offer new avenues for enhancing synaptic resilience and neuroprotection. Cutting-edge technologies, such as cryo-EM and single-cell sequencing, are expected to refine mechanistic insights into iGluR subtypes, while personalized medicine approaches integrating multi-omics with clinical trials could enable patient-tailored interventions. Finally, fostering international collaboration between high-output regions (e.g., U.S., China) and high-impact centers (e.g., Europe) will be essential to accelerate translational progress.

### Limitations

Although this study benefits from robust bibliometric tools and large databases, limitations still exist. First, our search strategy, while comprehensive within its scope, primarily relied on terminology directly related to “ionotropic glutamate receptors” and their subtypes. This approach may have inadvertently excluded relevant mechanistic studies that investigated broader synaptic glutamate dysregulation or excitotoxicity in AD without explicitly labeling them as iGluR-focused research. Second, reliance on citation metrics may overlook groundbreaking but less-cited work. Third, the adoption of a mixed statistical model fails to distinguish between original research and review articles. This undifferentiated approach substantially obscures the true value of original research, as existing evaluation systems rely mainly on citation counts, while review articles inherently receive higher citations due to their systematic summarization nature. There is an urgent need to develop next-generation bibliometric methods to establish classification-based statistical frameworks. Finally, the dynamic nature of scientific progress means that our analysis captures trends only up to the middle of 2025, necessitating periodic updates. Moreover, some analytical results can only reveal correlations (e.g., co-occurrence networks) but fail to elucidate the underlying causal relationships or deeper mechanisms.

### Conclusion

This bibliometric analysis maps the intellectual and collaborative landscape of iGluR research in AD, highlighting its evolution from mechanistic exploration to therapeutic innovations. Although NMDAR studies remain central, the field exhibits a mature and dynamically evolving profile. Future efforts should prioritize underexplored targets, such as AMPAR modulation, and leverage emerging technologies, including AI, cryo-EM, and single-cell sequencing, to refine therapies and expand clinical applications. By synthesizing these insights, our study provides a strategic roadmap for researchers, clinicians, and policymakers to allocate resources, intensify interdisciplinary collaboration, and accelerate the discovery of neurodegenerative disease interventions.
